# Popliteal Artery Aneurysm Repair in the Endovascular Era: Fourteen-Years Single Center Experience

**DOI:** 10.1097/MD.0000000000001130

**Published:** 2015-07-31

**Authors:** Sonia Ronchey, Felice Pecoraro, Vittorio Alberti, Eugenia Serrao, Matteo Orrico, Mario Lachat, Nicola Mangialardi

**Affiliations:** From the Department of Vascular Surgery, San Filippo Neri Hospital, Rome (SR, VA, ES, MO, NM); Vascular Surgery Unit, University of Palermo, AOUP “P. Giaccone”, Palermo, Italy (FP); and Clinic for Cardiovascular Surgery, University Hospital of Zurich, Zurich, Switzerland (ML).

## Abstract

To compare outcomes of popliteal artery aneurysm (PAA) repair by endovascular treatment, great saphenous vein (GSV) bypass, and prosthetic bypass.

Single center retrospective analysis of patients presenting PAA from 2000 to 2013. Patients were divided into endovascular treatment (group A); GSV bypass (group B); and prosthetic graft bypass (group C). Outcomes were technical success, perioperative mortality, and morbidity. Survival, primary and secondary patency, and freedom from reintervention rate were estimated. Differences in ankle-brachial index (ABI), in-hospital length of stay (InH-Los), red blood cell (RBC) transfusion, and limb loss were reported. Mean follow-up was 49 (median: 35; 1–145; SD 42) months.

Sixty-seven patients were included; 25 in group A, 28 in group B, and 14 in group C. PAA was symptomatic in 23 (34%) cases. Technical success was 100%. No perioperative death occurred. Three (4.5%) perioperative complications were reported with no significant difference between groups (*P* = 0.866). Five-years estimated survival was 78%. Estimated 5-years primary patency for groups A, B, and C was 71%, 81%, and 69%, respectively (*P* = 0.19). Estimated 5-years secondary patency for groups A, B, and C was 88%, 85%, and 84% (*P* = 0.85). Estimated 5-years freedom from reintervention for groups A, B, and C was 62%, 84%, and 70%, respectively (*P* = 0.16). A significant difference between preoperative ABI versus postoperative ABI was observed (*P* = 0.001). InH-LoS was significantly shorter in group A (*P* < 0.001). RBC transfusions were required significantly less in group A when compared to group C (*P* = 0.045). Overall limb salvage was achieved in all but 1 patient.

PAA repair has good early and long-term outcomes with different treatment options. Endovascular treatment was not inferior to surgical repair with a reduced InH-LoS and RBC transfusion. It can be successfully employed even in nonelective setting. A randomized controlled trial with long-term follow-up and appropriate patient inclusion criteria is necessary to compare these 3 treatment options.

## INTRODUCTION

Surgical bypass is considered the gold standard for popliteal artery aneurysm (PAA) repair, especially in young patients fit for conventional surgery.^1^ The great saphenous vein (GSV) is the ideal conduit and the prosthetic grafts a valid alternative to GSV for surgical bypass.^2^ Since the first endovascular treatment reported by Marin et al,^3^ a valuable increase in papers reporting on outcomes with this approach has emerged.^4^ This less invasive treatment allows the PAA exclusion also in patients considered unfit for conventional surgery. Mid and long-term results after PAA endovascular treatment are lacking. The aim of this study was to compare mid-term outcomes of endovascular treatment, GSV bypass and prosthetic bypass for PAA treatment in a single-center experience.

## METHODS

**FIGURE 1:**
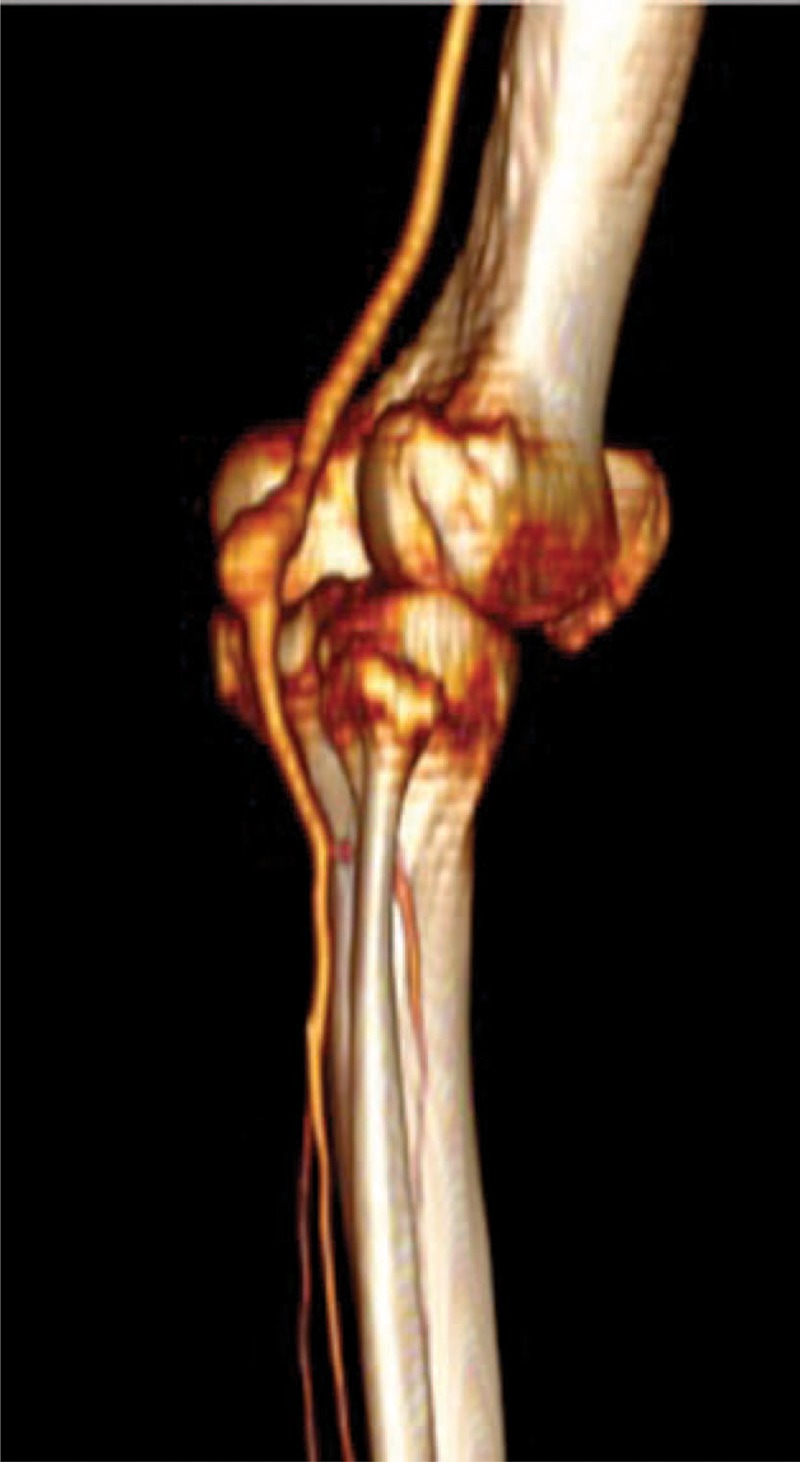
Preoperative CTA angiography three-dimensional reconstruction showing popliteal artey aneurysm location. CTA = computed tomography angiography.

From 2000 to 2013 data from patients treated for PAA were prospectively collected and retrospectively analyzed in January 2015. PAA endovascular treatment was introduced in our institution in 2000. All patients undergoing PAA endovascular treatment were included in the study. Control patients included more recent cases treated with GSV bypass and prosthetic bypass. Informed consent for the procedure was obtained from all patients and the study design was approved by Institutional Review Boards. Indications for PAA treatment were diameter > 20 mm or PAA related symptoms. For surgical PAA repair, the first option was GSV bypass; prosthetic grafts were employed when the GSV was not available/inadequate or in case of posterior access and for a very short bypass. Endovascular approach was employed in patients considered at high risk for conventional surgery at the beginning of our experience; from 2010 when the GSV was not available and if the anatomy was favorable for an endovascular treatment. The presence of proximal and distal landing zone of at least 1.5 cm was present in all endovascular procedures. In all cases, at least one run-off vessel was present before operation. All the included patients underwent a preoperative computed tomography angiography (CTA) (Figure 1). Patients were divided into 3 groups according to the treatment option: endovascular treatment (group A); femoro-popliteal bypass with GSV (group B); and femoro-popliteal bypass with prosthetic graft (group C). Heterogeneity between the 3 treatment groups was tested. Patients considered at high risk for conventional surgery and anatomically unsuitable for endovascular treatment were excluded from the study and treated by best medical treatment. Femoral artery aneurysm, posttraumatic PAA, and popliteal artery pseudoaneurysm were also excluded.

### Endovascular Technique

**FIGURE 2:**
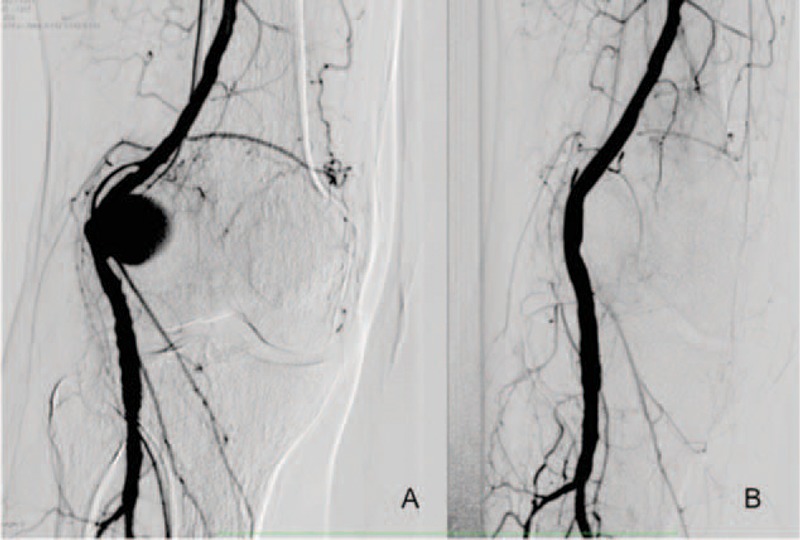
A, Intraoperative frontal view arteriogram. Popliteal artery aneurysm. B, Intraoperative frontal view arteriogram. Popliteal artery aneurysm exclusion after stent-graft deployment.

**FIGURE 3:**
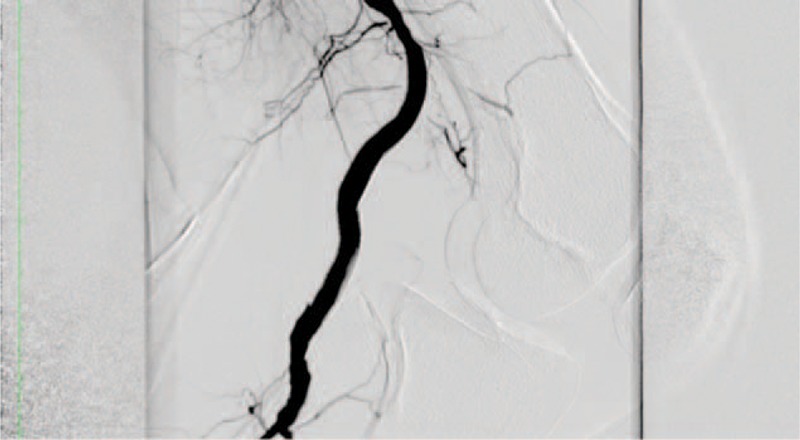
Intraoperative arteriogram. Lateral view with 90 joint flexion.

All endovascular procedures were performed in an operating room equipped with digital fluoroscopic C-arm (Euroampli ALIEN, Eurocolumbus, Italy) to allow a prompt conversion to open surgery if necessary. A vascular surgeon performed all procedures under general (5) or local anesthesia (20). In 17 cases access to PAA was omolater through a femoral cut-down; in the remaining 8 cases PAA was accessed percutaneously though the contralateral femoral artery. A weight-adjusted bolus of heparin was administrated intravenously and an intraoperative angiogram was performed to confirm the PAA location. In all cases the Viabahn Endoprosthesis (Gore, Flagstaff, Arizona) was employed to exclude the PAA. Stent-grafts were deployed with a minimum of 1.5 cm of proximal and distal landing zone (Figure 2). Stent-grafts size was chosen with 1 mm oversizing. A mean of 1.44 (r: 1–3; SD: 0.7) stent-grafts per patient was employed. A preoperative fibrinolysis was employed in 4 (16%) patients due to acute PAA thrombosis. A completion arteriography with knee in flection (>90°) was performed to assess the stent-graft flexibility (Figure 3). All patients underwent to dual antiplatelet regimen for at least 1 month.

### Surgical Technique

**FIGURE 4:**
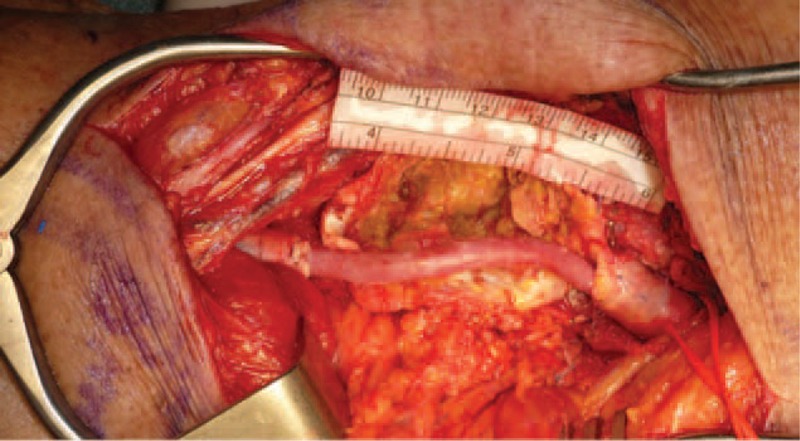
Intraoperative view of a short great saphenous vein bypass with posterior approach.

All procedures were performed under general anesthesia. A medial approach was chosen in 12 cases: these bypasses were performed according to the standard technique with surgical exposure of femoral bifurcation or proximal superficial femoral artery as inflow source. The distal popliteal artery was chosen as distal outflow point when possible; if not available the tibio-pernoeal trunk was the second option. For the medial approach, the bypass conduit was an autologous GSV in 10 cases and a prosthetic PTFE graft in 2 cases. The posterior approach was used in 30 cases and performed with patients in prone position using an S-shaped incision in correspondence of the popliteal fossa. The conduit for this approach was an autologous GSV in 18 cases and a prosthetic PTFE graft in 12 (Figure 4). The PTFE synthetic grafts employed were gelatin-coated PTFE (Vascutek Ltd, Renfrewshire, UK) grafts in 13 cases and heparin-bonded ePTFE (Propaten Gore-Tex; WL Gore & Associates Inc, Flagstaff, Ariz) graft in the remaining case.

### Follow-Up Protocol

All cases were followed with duplex ultrasound and clinical examination at 1, 6, and 12 months and annually thereafter. Endovascular cases underwent additionally to biplanar X-ray imaging before discharge.

## OUTCOMES

Early outcomes measured were technical success, perioperative mortality, and morbidity. Technical success was defined as procedure completed as intended. Late outcomes included survival, primary and secondary patency, and freedom from reintervention rate. All these outcomes were analyzed for the whole cohort and for each treatment group. Differences in preoperative, postoperative, and during follow-up were assessed for ankle-brachial index. In-hospital length of stay (InH-Los), red blood cell (RBC) transfusion, and limb loss were reported. Limb loss was defined as major amputation above or below the knee. Mean follow-up was 49 (median 35; 1–145; SD 42) months.

### Statistical Analysis

Means (*m*), median, range (*r*), and standard deviation (SD) were reported for parametric data; absolute values (n) and percentages (%) for nonparametric data. Differences between groups were assessed using the t test or ANOVA for parametric data and the *χ*^2^ test for categorical variable. Kaplan–Meier curves were used to estimate survival, primary and secondary patency, and freedom from reintervention. Differences in curves were assessed with the Brelow test. Statistical significance was assigned at *P* < 0.05. Statistical analysis was performed using SPSS16.0 (SPSS Inc, Chicago, IL).

## RESULTS

A total of 67 nonconsecutive patients (55 males) were included, with a mean age of 69 (median 69; *r*: 49–87; SD: 9) years. According to the treatment option, 25 patients were assigned to group A, 28 to group B, and 14 to group C. Indication to PAA treatment was symptoms presence in 23 (34%) cases; of these 14 (61%) presented an acute PAA symptoms onset (4 in group A; 8 in group B, and 2 in group C; *P* = 0.72). Demographics and comorbidities are reported in Table 1.^5–7^ Mean PAA diameter was 28 (median: 26; *r*: 22–54; SD: 8) mm with no significant differences between groups (24 vs. 27 vs. 26; *P* = 0.26). In the group A mean diameter at proximal and distal landing zone was 5.4 (SD: 2) mm and 4.5 (SD: 1) mm, respectively. The heterogeneity test with multiple comparisons between the 3 groups showed a significant difference in the age variable for group A versus B (*P* = 0.002); and in chronic obstructive pulmonary disease for group A versus B (*P* = 0.026) and for group A versus C (*P* = 0.010). All other examined parameters, including acute onset of symptoms and preoperative run off vessels, showed no significant differences (Table 1). Mean preoperative ABI was 0.72 (median: 0.6; SD: 0.3); no statistical significant differences were observed between groups A, B, and C (0.69 vs. 0.72 vs. 0.73; *P* = 0.10).

**TABLE 1:**
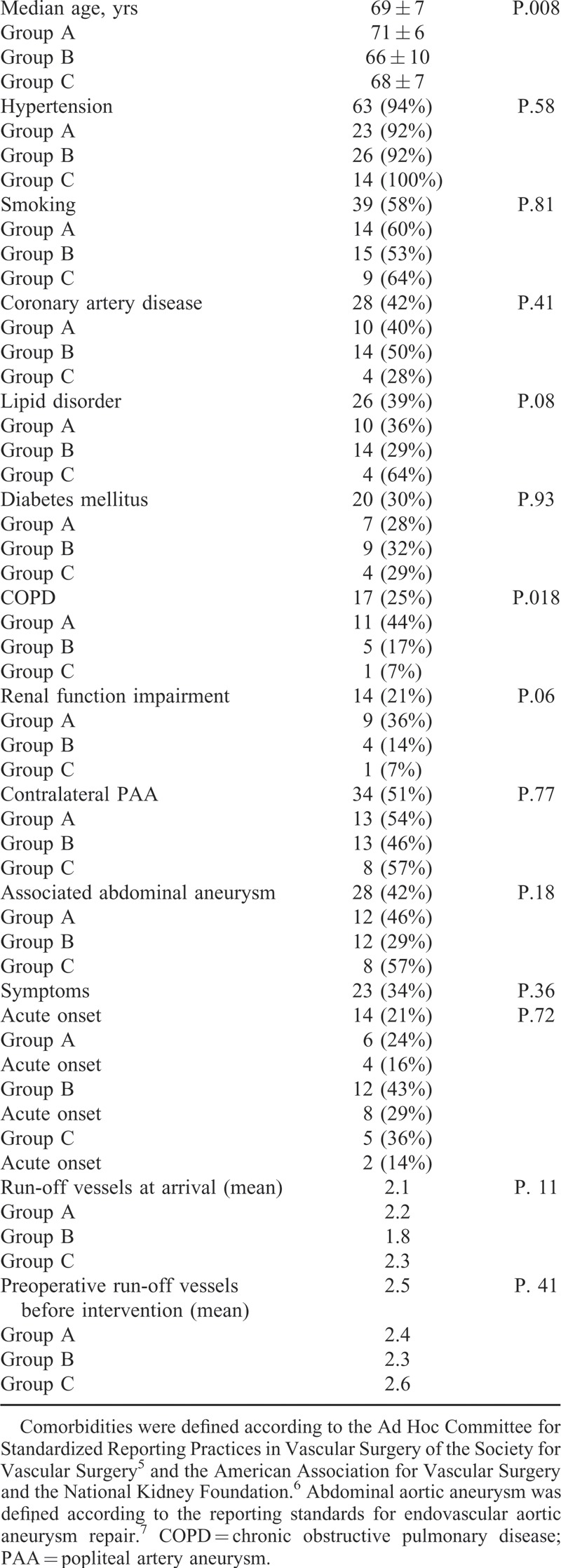
Heterogeneity Test for Demographics and Comorbidities by Groups

In 16 patients a preoperative intra-arterial thrombolysis was employed before PAA treatment, in 4 cases of the group A, in 10 of the group B, and in 2 of the group C. The intra-arterial thrombolysis was used for all acute symptomatic PAA (14/14; 100%) patients and in 2 nonacute symptomatic (2/23; 9%) PAA patients of the group B.

Technical success was achieved in all cases. No perioperative death occurred in all groups. Overall 3 (4.5%) perioperative complications were observed in 3 patients: 1 (4%) patient in group A presented to inguinal hematoma; 1 (4%) in group B and 1 (7%) in group C had a wound bleeding requiring surgical hemostasis. No significant difference in complication rate was observed between the 3 groups (*P* = 0.866). Mean postoperative ABI was 0.89 (median: 0.9; SD: 0.15); between groups a statistical difference in favor of group A was observed in comparison to group B and C (0.95 vs. 0.85 and 70.9; *P* = 0.022).

**FIGURE 5:**
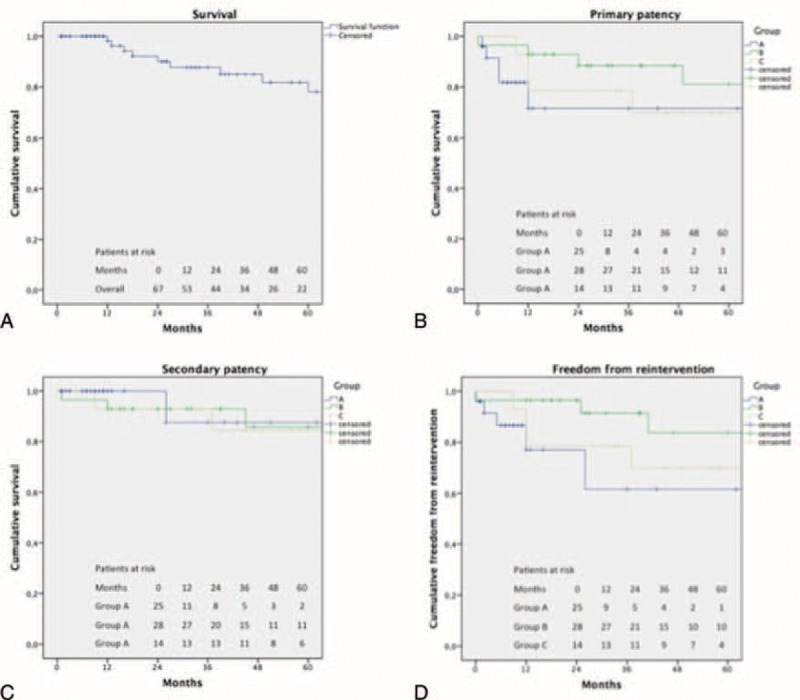
A, Overall cumulative survival. Standard error (SE) did not exceed 10% at 60-month follow-up (SE = 8% at 60 months). B, Primary patency. SE did not exceed 10% at 60-month follow-up for group B (SE = 9% at 60 months). SE exceed 10% at 12 months in group A (SE = 12% at 60 months) and C (SE = 13% at 60 months). C, Secondary patency. SE did not exceed 10% at 60-month follow-up for group B (SE = 8% at 60 months). SE exceed 10% at 26 months in group A (SE = 12% at 60 months) and at 37 months in group C (SE = 10% at 60 months). D, Freedom from reintervention. SE did not exceed 10% at 60-month follow-up for group B (SE = 9% at 60 months). SE exceed 10% at 12 months in group A (SE = 16% at 60 months) and C (SE = 13% at 60 months).

Overall, 5 years estimated survival was 78% (Figure 5A). During follow-up overall graft thrombosis occurred in 14 (21%) patients: 5 (20%) occlusion in group A; 5 (19%) in group B, and 4 (29%) in group C. No significant difference in occlusion incidence between the 3 groups (*P* = 0.726) was observed during the follow-up. No stent-graft fracture was observed in group A. Mean interval time to thrombosis was 18 (median: 6; *r*: 0–73; SD: 21) months.

Estimated 5-years primary patency for groups A, B, and C was 71%, 81%, and 69%, respectively, with no statistical differences between the 3 treatment groups (*P* = 0.19) (Figure 5B). Estimated 5-years secondary patency for groups A, B, and C was 88%, 85%, and 84% with no statistical differences between curves (*P* = 0.85) (Figure 5C). During the follow-up all secondary interventions were occlusion-related. These were necessary in 12 of the 14 occlusions; 4 were performed in group A, 5 in group B, and 3 in group C. No statistical significant differences were observed in reintervention rate between the 3 groups (*P* = 0.918). The remaining 2 patients presenting a graft thrombosis underwent to limb amputation for irreversible ischemia and medical therapy for an asymptomatic occlusion. Estimated 5-years freedom from reintervention for groups A, B, and C was 62%, 84%, and 70%, respectively, with no statistical differences between curves (*P* = 0.16) (Figure 5D). At mean follow-up ABI was 0.87 (median: 0.9; SD: 0.16) and no statistical significant differences were observed between groups A, B, and C (0.82 vs. 0.75 vs. 0.79; *P* = 0.13). Overall a significant difference was observed between preoperative ABI versus postoperative ABI (*P* = 0.001), and preoperative ABI versus follow-up ABI (*P* = 0.003). No differences were observed between postoperative ABI versus follow-up ABI (*P* = 0.18)

Mean InH-LoS was 6.5 (median: 7; *r*: 2–14; SD: 3) days. InH-Los was 4.1 (median: 4; SD: 1) days, 7.9 (median: 8; SD: 2) days, and 8.01 (median: 8; SD: 2) days for groups A, B, and C, respectively; the multiple comparisons between the 3 groups showed a significant difference InH-LoS for group A versus B (*P* < 0.001) and for group A versus C (*P* < 0.001). RBC transfusion was required in 1, 6, and 4 patients in groups A, B, and C, respectively; the multiple comparison between the 3 groups showed a significant difference in RBC transfusion for group A versus group C (*P* = 0.045). Overall limb salvage was achieved in all but 1 patient presenting symptoms. During follow-up this patient from group B underwent to below the knee amputation after 2 years due to bypass occlusion. No limb loss was observed in patients presenting asymptomatic PAA.

## DISCUSSION

PAAs are the most common peripheral aneurysms with a reported incidence of 0.1–3%. In asymptomatic patients, to prevent potential complications, the reported indications to PAA repair are size >20 mm, high-grade thrombus and poor run-off vessels. PAA symptoms represent an absolute indication to repair.^8,9^ Some authors justify an aggressive PAA management for size less than 20 mm due to a reported amputation rate of 30% after PAA acute thrombosis.^10^ Also compartment syndrome and peripheral embolization are PAA complications with an increased limb loss risk leading, infrequently, to death.^11–14^ Also PAA rupture is rare and 50–75% of these cases present with pain and swelling in the popliteal fossa and symptoms of lower extremity ischemia.^15^ In the present series PAA symptoms were the indication to repair in 34% of cases; 6 (26%) of these 23 patients were in group A.

PAA instrumental diagnosis is easily achieved with duplex ultrasound (DUS) test that can differentiate PAA from other popliteal fossa pathologies such us Baker cysts or tumors. Computed tomographic angiography (CTA), magnetic resonance angiography, and/or invasive arteriography are necessary to plan an endovascular treatment.^16^ In all cases PAA diagnosis was achieved with DUS. In group A, patients underwent additionally to CTA in order to assess PAA anatomical findings and the feasibility of endovascular treatment.

PAA surgical treatment is burdened by a relatively low perioperative mortality rate of 0–1% in asymptomatic patients and about 2% in symptomatic cases; conversely, a high perioperative morbidity mainly related to access complications is reported ranging from 30% to 40%.^17^ The PAA endovascular treatment has shown excellent perioperative outcomes in terms of mortality and morbidity.^18^ Potential complication of endovascular treatment, such as endograft fracture, endoleak, or migration, has been rarely reported.^19–22^

The endovascular approach was introduced as an alternative in patients unfit for conventional surgery. Galinanes et al have reported the increase of this less invasive intervention after 10 years from its introduction. In this review of the Centers for Medicare and Medicaid inpatient claims between 2005 and 2007, endovascular interventions have significantly increased (11.7% vs. 23.6%, *P* < 0.0001).^23^ Despite this, the small-size long-term results, the limited experience reported, and the reduced patency rate have been considered limitations of this technique.^24^

The use of Haemobahn/Viabahn stent-graft (W.L. Gore & Associates Inc., Flagstaff, AZ, USA) has been advocated as first option stent-graft due to the PTFE cover and the relative flexibility in order to increase patency outcomes. However, diameter discrepancy from proximal to distal landing zone represents a relevant limitation of this device.^25^

Also anatomic features of popliteal artery with regards to physiological knee movements determining artery morphological changes and stent stress represent adjunctive limitations for a stent implantation. These morphological alterations are deemed responsible for the reduced patency rate after stent placement, which is the Achilles tendon of PAA endovascular treatment.^26^ The use of a contralateral access in percutaneous endovascular PAA repair was chosen to reduce access site complication risk. In fact, the use of large introducer in an antegrade fashion has been related to an increased risk of femoral access complication. When a percutaneous contralateral access was not feasible due to anatomic limitations (such as angulated aorta or iliac stenosis), an omolateral femoral cut-down was employed. For surgical treatment, the bypass conduit choice has been historically related to patency outcomes. In a Mayo Clinic study of 385 surgical PAA treatments, the 5-year primary and secondary patency rates were 76% and 87%, respectively; these outcomes were significantly better with GSV (85% and 94%) when compared to PTFE prosthetic graft (50% and 63%) (*P* < 0.05).^27^ Similar results have been reported with the posterior approach.^9^ In the present series, we did not observe a significant difference in the 5-years primary and secondary patency outcomes between GSV and PTFE prosthetic graft. Piazza et al, in a single-center experience with endovascular repair of 47 PAA, report a 5-year primary and secondary patency of 76% and 82%, respectively.^28^ These results are comparable with those reported in the present study with a 5-years primary and secondary patency of 69% and 84%, respectively, after endovascular repair. During last few years, no inferior results have been reported of endovascular PAA repair when compared to bypass surgery in terms of patency.^29^ We have not found significant differences between the 3 treatment groups in terms of primary and secondary patency, but a tendency for better primary patency outcomes in group B of GVS bypass. In a retrospective multicentric study on 312 PAAs, also Pulli et al reported no significant differences between open (178 PAAs) and endovascular (134 PAAs) repair in terms of primary and secondary patency.^18^ The primary patency outcome was clinically reflected by the ABI result: overall ABI analysis reported a significant improvement from preoperative to postoperative stage. This advantage was durable at follow-up analysis. However, a nonsignificant slight decrease in ABI values was observed in group A from postoperative to follow-up stage in accordance with the nonsignificant decrease in patency outcome.

As already reported,^17^ a significant advantage in terms of InH-LoS is evident with an endovascular approach; moreover, we have also found a significant advantage also in terms of RBC transfusions.

Patient age has been related to popliteal artery tortuosity with an increasing degree proportional to age,^30^ especially in the supra-genicular segment.^17^ Despite this, and according to most authors recommendations,^31^ we have used the endovascular approach in older patients unfit for conventional PAA vein bypass treatment. A recent decision analysis study, with Markov model, suggests that GSV bypass is still the PAA treatment gold standard. Patients with short life expectancy or with advanced age should undergo to conservative management. Thus, endovascular approach is indicated for high-risk patients not suitable for open repair and for patients without a suitable autologous vein.^1^ Also, our PAA protocol provided the GSV bypass as first line approach and the prosthetic bypass as a second option. However, from 2010, a shift toward the endovascular PAA repair as second-option treatment from the prosthetic bypass was observed. The lack of guidelines^32^ and evidences to advocate a change in the PAA “gold standard” surgical treatment^1^ is evident. A randomized controlled trial with a large homogeneous population is awaited.^33^ In our experience patients treated endovascularly were significant elderly, with higher rate of COPD and there was tendency of worst renal function. Despite this, the endovascular treatment was not inferior in terms of early outcomes (technical success, perioperative mortality, and morbidity) and mid-term outcomes (survival, primary and secondary patency, and freedom from reintervention rate) when compared to surgical options, but a reduced InH-Los and RBC transfusion were reported.

Limitations of this study are the lack of randomization, the retrospective analysis, and the inclusion criteria of the control groups. Moreover to the lack of significant difference for outcomes such as primary patency can be related to the relative small patient sample.

## CONCLUSION

PAA repair is safe with good early and long-term results independently from the treatment option adopted. The endovascular treatment has anatomic limitations related to popliteal artery movements but is not inferior to surgical prosthetic graft repair. This approach has a reduced InH-LoS and RBC transfusion and it can be successfully employed in nonelective setting. The GSV has nonsignificantly better outcomes in comparison to prosthetic graft. A randomized controlled trial with long-term follow-up and appropriate patient inclusion criteria is necessary to compare the 3 treatment options.
